# The Open Brain Consent: Informing research participants and obtaining consent to share brain imaging data

**DOI:** 10.1002/hbm.25351

**Published:** 2021-02-01

**Authors:** Elise Bannier, Gareth Barker, Valentina Borghesani, Nils Broeckx, Patricia Clement, Kyrre E. Emblem, Satrajit Ghosh, Enrico Glerean, Krzysztof J. Gorgolewski, Marko Havu, Yaroslav O. Halchenko, Peer Herholz, Anne Hespel, Stephan Heunis, Yue Hu, Chuan‐Peng Hu, Dorien Huijser, María de la Iglesia Vayá, Radim Jancalek, Vasileios K. Katsaros, Marie‐Luise Kieseler, Camille Maumet, Clara A. Moreau, Henk‐Jan Mutsaerts, Robert Oostenveld, Esin Ozturk‐Isik, Nicolas Pascual Leone Espinosa, John Pellman, Cyril R Pernet, Francesca Benedetta Pizzini, Amira Šerifović Trbalić, Paule‐Joanne Toussaint, Matteo Visconti di Oleggio Castello, Fengjuan Wang, Cheng Wang, Hua Zhu

**Affiliations:** ^1^ Radiology Department CHU Rennes Rennes France; ^2^ Inria, CNRS, Inserm, IRISA UMR 6074, Empenn ERL University of Rennes Rennes France; ^3^ Department of Neuroimaging King's College London London United Kingdom; ^4^ Memory and Aging Center, Department of Neurology University of California San Francisco California USA; ^5^ Dewallens & partners law firm, Leuven, Belgium & Antwerp Health Law and Ethics Chair (AHLEC) and P^2^ research group, Faculty of law University of Antwerp Antwerp Belgium; ^6^ Ghent Institute for functional and Metabolic Imaging Ghent University Ghent Belgium; ^7^ Oslo University Hospital Oslo Norway; ^8^ McGovern Institute for Brain Research Massachusetts Institute of Technology, Cambridge, MA, USA; Department of Otolaryngology ‐ Head and Neck Surgery, Harvard Medical School Boston Massachusetts USA; ^9^ Department of Otolaryngology ‐ Head and Neck Surgery Harvard Medical School Boston Massachusetts USA; ^10^ Aalto University Espoo Finland; ^11^ International Laboratory of Social Neurobiology, Institute of Cognitive Neuroscience, National Research University Higher School of Economics Moscow Russia; ^12^ Stanford University Stanford California USA; ^13^ Department of Neuroscience and Biomedical Engineering Aalto University Espoo Finland; ^14^ Dartmouth College Hanover New Hampshire USA; ^15^ NeuroDataScience ‐ ORIGAMI laboratory, McConnell Brain Imaging Centre, The Neuro (Montreal Neurological Institute‐Hospital), Faculty of Medicine McGill University, Montreal Quebec Canada; ^16^ CHU Rennes France; ^17^ Eindhoven University of Technology Eindhoven The Netherlands; ^18^ Institute for Experimental Psychology Heinrich‐Heine‐University of Düsseldorf Düsseldorf Germany; ^19^ School of Psychology Nanjing Normal University Nanjing China; ^20^ Erasmus University Rotterdam Rotterdam The Netherlands; ^21^ Biomedical Imaging Unit FISABIO‐CIPF Foundation for the Promotion of Health and Biomedical Research of the Valencian Community Valencia Spain; ^22^ Department of Neurosurgery, St. Anne's University Hospital Masaryk University Brno Czech Republic; ^23^ Department of Advanced Imaging Modalities, MRI Unit, General Anti‐Cancer and Oncological Hospital of Athens“St. Savvas” National and Kapodistrian University of Athens Athens Greece; ^24^ Department of Neurosurgery and Neurology National and Kapodistrian University of Athens Athens Greece; ^25^ Department of Psychological and Brain Sciences Dartmouth College Hanover New Hampshire USA; ^26^ Inria, University of Rennes, CNRS Inserm, IRISA UMR 6074, Empenn ERL U 1228 Rennes France; ^27^ Pasteur Institute Paris France; ^28^ Department of Radiology and Nuclear Medicine Amsterdam University Medical Centers Amsterdam The Netherlands; ^29^ Department of Radiology and Nuclear Medicine University Hospital Ghent Ghent Belgium; ^30^ Donders Institute for Brain Cognition and Behaviour; Radboud University Nijmegen The Netherlands; ^31^ Bogazici University Istanbul Turkey; ^32^ Biomedical Imaging Unit FISABIO‐CIPF, Foundation for the Promotion of Health and Biomedical Research of the Valencian Community Valencia Spain; ^33^ Zuckerman Mind Brain Behavior Institute Columbia University New York New York USA; ^34^ Centre for Clinical Brain Sciences University of Edinburgh Edinburgh United Kingdom; ^35^ University of Verona Verona Italy; ^36^ Faculty of Electrical Engineering University of Tuzla Tuzla Bosnia and Herzegovina; ^37^ McGill University Montreal Neurological Institute and Hospital, Montreal Quebec Canada; ^38^ Helen Wills Neuroscience Institute University of California Berkeley California USA; ^39^ National Institute of Education Nanyang Technological University Singapore Singapore; ^40^ School of Health Fujian Medical University Fuzhou China; ^41^ Department of Biological Medicine and Engineering, BUAA Beihang University Beijing China

**Keywords:** brain imaging, general data protection regulation, informed consent

## Abstract

Having the means to share research data openly is essential to modern science. For human research, a key aspect in this endeavor is obtaining consent from participants, not just to take part in a study, which is a basic ethical principle, but also to share their data with the scientific community. To ensure that the participants' privacy is respected, national and/or supranational regulations and laws are in place. It is, however, not always clear to researchers what the implications of those are, nor how to comply with them. The Open Brain Consent (https://open-brain-consent.readthedocs.io) is an international initiative that aims to provide researchers in the brain imaging community with information about data sharing options and tools. We present here a short history of this project and its latest developments, and share pointers to consent forms, including a template consent form that is compliant with the EU general data protection regulation. We also share pointers to an associated data user agreement that is not only useful in the EU context, but also for any researchers dealing with personal (clinical) data elsewhere.

## 1 GOAL AND BACKGROUND

Petabytes of brain imaging data are collected for research purposes every year, yet only a small fraction becomes publicly available despite evidence for the benefits of sharing such data sets (Milham et al., 2018). One reason, among others, is that openly sharing human brain imaging data requires conforming to established ethical and legal norms, in particular with respect to ensuring that research participants' privacy is respected. Ethical and legal requirements are usually validated by institutional review boards (also known as research ethics committees), which operate under national, federal, and/or supra‐national regulations. In the case of brain imaging, ethical and legal norms generally follow international recommendations for medical research involving human participants, in particular those from the World Medical Association: the declaration of Helsinki (World Medical Association, 2001) which lays down ethical principles for medical research involving human subjects, and the declaration of Taipei (World Medical Association, 2017) which lays down ethical principles regarding health databases and biobanks.

In some scientific disciplines, for example, genetics (Khan, Capps, Sum, Kuswanto, & Sim, 2014), consent is widely discussed and analyzed, and templates for participant consent forms are available and commonly used, for example, for clinical trials (https://www.who.int/ethics/review-committee/informed_consent/en/). To date, similar work has not been undertaken for brain imaging studies. The goal of the Open Brain Consent initiative is to facilitate brain imaging data sharing by providing practical tools that enable data sharing while respecting research participants' privacy. It consists primarily in providing widely acceptable information/consent forms allowing processing and deposition of data into appropriate archives for future (re)use. Additionally, the project website references tools/pipelines to minimize the risk of re‐identification and provides additional information about the various regulations to help brain imaging researchers.

## 2 PROJECT HISTORY AND CONTRIBUTION MODEL

The Open Brain Consent project was started in 2014 to provide (a) a collection of existing samples of consent forms allowing data sharing, (b) a reference “ultimate” consent form, and (c) tools helpful for pseudonymization, making brain imaging data easier to share. The goal of having a template consent form was, and still is, to establish a recommended wording for a consent form based on collected examples that represent community wide expertise. At that time, the OpenfMRI archive (later developed into OpenNeuro) (Poldrack et al., 2013) was confronted with issues related to the rights to share the growing number of data sets being submitted. To address them, OpenfMRI established a recommended wording which was contributed to the Open Brain Consent project in 2015. Since then, many researchers have joined the project to provide translations to a number of languages and to expand the list of sample forms and tools. In 2018, the advent of the European General Data Protection Regulation (GDPR: https://gdpr-info.eu) left many researchers unsure about the sharing of brain imaging data, since anonymous data can be shared freely, but personal data cannot. An online discussion ensued concerning the status of brain imaging data, and work began to revise the “Ultimate” Open Brain Consent form to make sharing brain imaging data, GDPR compliant. This work took place in particular during the Organization for Human Brain Mapping (https://www.humanbrainmapping.org) “hackathon” in Rome (June 2019). Based on this work, the most recent rewriting took place in November 2019 (and the following weeks) during a GLiMR action workshop (https://glimr.eu) hosted at the COST association (https://www.cost.eu) in Brussels.

The Open Brain Consent project is hosted on GitHub (https://github.com/con/open-brain-consent). Contributions to the project are submitted via GitHub's Pull Request mechanism for changes to the text and recommended additions to sample forms or detected issues are proposed via Issues. The project is open access, all materials are provided under CC‐BY‐SA 3.0 license, and we encourage researchers across the world to contribute their knowledge about data privacy, (personal) information protection, data sharing and consent. The full history of changes to the project is available in its Git history, and citable releases are provided through Zenodo.org (Halchenko et al., 2019).

## 3 ETHICAL CONCERNS WHEN SHARING BIOMEDICAL AND BRAIN IMAGING DATA

As more brain imaging data and biomedical data are shared openly, concerns have been raised in several publications about risks to data privacy. From a legal and ethical standpoint, risks about research participants' privacy must be identified and mitigated. This necessitates, on one hand, that procedures for data de‐identification are in place (from pseudonymization to full anonymization) along with means for individuals to exercise control over the use of their personal data. On the other hand, it requires retaining as much as possible information in the data, allowing researchers to use the data to answer specific research questions. Thus, a balance needs to be struck and that balance is influenced, in part, by the risks of re‐identification based on current technological possibilities and limitations. For instance, it has been shown that it is possible to identify participants in the 1,000 Genomes Project by combining publicly available demographic information from the American census and public information from the peoplefinder.com website with anonymized genomic data sets (Gymrek, McGuire, Golan, Halperin, & Erlich, 2013). This work, however, relied on having been given secured access to the genomic data and being able to code and use advanced cryptographic algorithms; hence, it can be argued that the risk of identification remains low. By contrast, Rocher, Hendrickx, and de Montjoye (2019) (Rocher et al., 2019) estimated the likelihood of re‐identification of individuals at around 95% by combining biomedical data and information from postcodes and census using relatively simple statistical models available in open source packages like R or Python. The cost and know‐how, in that case, is low and the risk of re‐identification is thus higher.

**FIGURE 1:**
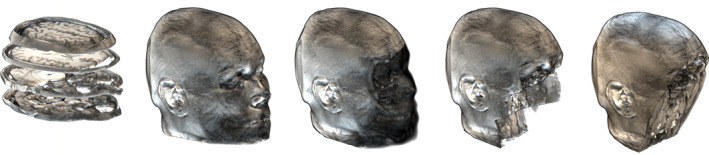
The typical structural MRI of the brain is made up of a series of 2D slices (left) from which it is easy to reconstruct a face. Pseudonymization procedures (from the middle to right) go from blurring/masking the face to zero‐out an entire part of the image, increasing anonymity but decreasing usage and sometimes damaging the frontal part of the brain. (This image was made from the MRI of one of the authors, CP, visualized with MRICRoGL, masked using mask_face (https://nrg.wustl.edu/software/face‐masking/usage/), mri_deface from the freesurfer suite (https://surfer.nmr.mgh.harvard.edu/fswiki/mri_deface) and SPM12 (https://www.fil.ion.ucl.ac.uk/spm/software/spm12/) — (https://doi.org/10.7488/ds/2877)

Brain imaging data are often collected along with a range of associated biomedical and/or clinical data which represent additional identifying features. Even if additional biomedical data are not provided, there are brain imaging specific concerns, especially for magnetic resonance imaging (MRI) data. From a standard anatomical MRI of the participants' head, the facial features can be reconstructed in 3D and matched to publicly accessible photos. Various approaches have been proposed to “deface” MRI data, from blurring to zeroing (some e.g., of defacing algorithms are presented in Figure 1). Such approaches cause data loss and, if performed too coarsely, can affect the outcome of analysis pipelines (de Sitter et al., 2020). In addition, recent advances in machine learning have cast doubt on the efficacy of this approach. Abramian and Eklund (2019) have been able to “reface” single slice data with relative success (~60 to 75% success) using machine learning (employing a Generative Adversarial Network), and it is reasonable to anticipate that methods like these will improve and become more widely available in the future. Beyond re‐identification using direct identifiers, GDPR highlights that singling out is a precondition to identification, and it should therefore be minimized. Identification can be straightforward with an anatomical MRI in which the face is available since faces are likely unique (Sheehan & Nachman, 2014), but singling‐out individuals from defaced data is also possible based on the gyral patterns that are unique to every individual (Duan et al., 2020), like fingerprints. From MRI data that do not include facial information or detailed anatomy, such as functional MRI data, it is still possible to single out individuals. For example, Ravindra and Grama (2019) were able to single out participants across multiple data sets, using task performance and connectivity patterns, with a success of ~90%. Altogether, these results suggest that biomedical data and brain MRI in particular, are at risk of re‐identification—that is, can in all likelihood not be fully anonymized—and should therefore be considered as personal data under the GDPR. Acknowledging that risks to personal data privacy exist for brain imaging data, identifying them and putting mechanisms in place to mitigate them are therefore essential, as is informing each participant throughout the process: these are core steps in the Open Brain Consent working group.

## 4 THE ULTIMATE CONSENT FORM

Provided that national regulations allow data sharing in open public databases, a consent form template for openly sharing brain imaging data have been established, and is available in seven languages (Chinese, English, French, German, Italian, Polish, Spanish—https://open-brain-consent.readthedocs.io/en/stable/ultimate.html). This template has been established before the GDPR was in place, and is recommended for researchers outside the EU. It was informed by existing consent forms from various institutions and from discussions with ethicists. As discussed above, it aims to (a) provide privacy‐related information to the participants and (b) secure open data sharing for researchers. It also establishes a difference between the consent to take part in a research study, and the consent for sharing data for secondary usage, while these can still be combined in a single information notice.

Another feature on the consent form is that it comes in two “flavors” with a single versus dual access model. This differentiates the open and public sharing of all versus some of the data. In the latter case, researchers can give controlled access to the data not publicly shared. This is necessary to address privacy issues related to sharing biomedical metadata which increases risk of re‐identification, as discussed above.

## 5 THE OPEN BRAIN CONSENT, GDPR EDITION

Under the European GDPR, two types of data are defined: anonymous and personal data (Mourby et al., 2018), the latter being further subdivided based on its sensitivity. Personal sensitive data are data revealing racial or ethnic origin, sexual orientation, political opinions, religious, or philosophical beliefs, trade union membership, genetic data, biometric data processed solely to identify a human being, and health data. In this context, pseudonymization is a procedure to reduce the risk of identification by removing or replacing individual identifiers—for example, address, name—while retaining those identifiers separately from the rest of the individual information (i.e., with restricted access), thus making it difficult but not impossible to retrace this information to the actual subject. Since pseudonymization does not entirely delete the link between the information and the individual, this does not change the status of the data from personal to anonymous according to the GDPR, thus GDPR does not recognize pseudonymized data as a distinct category. This means that even after removing direct identifiers such as names, addresses, but also facial features, MRI data are likely to remain classified as personal data, since there is still a risk of re‐identification. Such a classification in turn requires compliance with all relevant aspects of GDPR.

The GDPR‐compliant template form (https://open-brain-consent.readthedocs.io/en/stable/gdpr/index.html) was taken from the ultimate Open Brain Consent form and adapted to comply with the GDPR, using examples from existing privacy statements and participant information letters encountered by members of our working group. The key elements are to (a) have a consent form that only deals with data sharing; (b) inform participants about the data storage, privacy measures (e.g., pseudonymization procedure) and control over usage (e.g., withdrawal) and; (c) provide information on how data will be shared, specifically outside the EU. These key elements must be included to promote secondary use of the data (Staunton, Slokenberga, & Mascalzoni, 2019). The main difference with the non‐EU specific consent form is that further information about privacy and usage control is provided. For researchers from the EU and affiliated countries, we therefore recommend having, in addition to their study consent form, a separate data sharing consent form based on this template.

### 5.1 Data user agreement

As part of information on how data will be shared, we recommend using a data user agreement (DUA) rather than a license, and a template DUA is also provided. Both, the consent and the DUA, are available in 11 languages (Bosnian, Czech, Dutch [NL/BE], English, Finnish, French, Italian, Norwegian, Greek, Spanish, Turkish—https://open-brain-consent.readthedocs.io/en/stable/gdpr/data_user_agreement.html). Since brain imaging data are seen as personal data, they are protected and sharing cannot be open and public without a legal ground/lawful basis under GDPR, and therefore only one type of access is proposed. The use of a DUA is recommended to help mitigate risks to personal data privacy of the research participants, while still supporting the sharing of said data with the wider research community. The proposed DUA explicitly asks the applicant—the researcher applying to access the participant data—to confirm that they will refrain from redistributing the data and attempting to re‐identify the participants. It also makes it clear that any applicant who downloads the data becomes the data controller, a natural or legal person, who alone or jointly with others, determines the purposes and means of the further processing of the personal data. This new data controller is then responsible for the appropriate usage of the copy of the shared data, and for ensuring that the agreed terms and conditions are applied/taken care of. This new data controller—or applicant—does not have to be within the EU, but agrees with the DUA—which refers to the Standard Contract Clauses (https://ec.europa.eu/info/law/law-topic/data-protection/international-dimension-data-protection/standard-contractual-clauses-scc_en) approved by the European Commission for data transfers to data controllers outside the EU, thus complying with the GDPR—by signing it. Licenses, in contrast, do not impose such restrictions. While a DUA must be signed, and usage is limited, it still allows for easy access and broad reuse within the scientific community. Our proposal is for institutions to have a “click‐through” DUA or similarly automated system rather than having ad hoc decisions on a case by case basis, which stands against modern open data practices. This would be particularly important/ethically desirable if researchers who collect data are also the ones deciding who has access to them (Bishop, 2016). Having said that, there are also practical and legal reasons for not using automated systems, for example, how to ensure the identity of a signatory of the DUA. If the DUA is not correctly signed by a duly identified controller, then this may render the DUA legally invalid. There are, however, solutions to this as well, for example, using electronic signatures or registered user accounts.

## 6 DISCUSSION

The Open Brain Consent project aims at facilitating human brain imaging data sharing. By sharing these data as openly as possible, researchers are confronted with ethical and legal issues. While ethical issues are internationally recognized and discussed, they are legally translated differently across countries creating confusion. Here we tried to reconcile these two aspects by offering two generic consent template forms that should help with the law in most situations.

Recent technological advances, not only in gathering data and linking databases, but also from statistical modeling and machine learning, increase the risk of re‐identification of pseudonymized data. As a result, it is essential to provide up‐to‐date information to research participants about data privacy (both privacy risks and right of control) which are included in the consent forms. Within the EU context, data that were previously thought to be anonymous are now considered personal. Although pseudonymization of biomedical data is still necessary and encouraged, it does not change the data status from personal to anonymous. Thus, compliance with the GDPR is required and, depending on national regulation, secured access (with or without a DUA) might be necessary. We provide information/consent templates and a DUA template for these different cases, which we believe will improve researchers' likelihood of getting approval from their institutional review boards/ethics committees to share brain imaging data on web‐serviced data repositories.

More recent data platform technologies rely on distributed data storage and/or processing models. A data set collected at multiple sites could be stored and processed at multiple locations, and yet accessed via a single query given a user is authorized to access the data (see e.g., http:// datalad .org). It remains to be seen how a DUA could be implemented for such a distributed model. In other cases, data analysis can be performed (with local or remote execution) using algorithms implementing federated learning (Sheller et al., 2020) and differential privacy concepts (redaction threshold, noise addition, query limitations, Plis et al., 2016). In such scenarios, privacy concerns are greatly reduced and the consent template should be modified accordingly, in particular regarding data confidentiality. Finally, other initiatives rely on local data processing and sharing of aggregate/derivative data only (Plis et al., 2016; Thompson et al., 2014). If individuals cannot be singled out in the shared results, a DUA is not necessary since raw/individual data remain with the data processor and re‐identification becomes impossible.

While we believe standardized templates such as these from the Open Brain Consent working group play an important role in advancing transparent research practices, they do not provide a complete solution to the complex challenges involved in sharing research data. For example, are data from brain imaging techniques other than MRI also at risk or re‐identification? Since many brain imaging data sets include various demographics, clinical metadata, and perhaps even multimodal imaging data, these are likely at risk too. As noted earlier, structural MRI data are at high risk of re‐identification because facial features are available if not sufficiently removed or obscured. Since functional MRI can also be used to single out individuals (Ravindra & Grama, 2019) despite not having such defining features, it seems pertinent to extrapolate this possibility to other whole‐brain imaging techniques, for example, magneto‐ or electro‐ encephalography (MEG‐EEG). In fact, previous work has demonstrated that a simple EEG event‐related potential (ERP) from a single electrode has a discriminability d‐prime of around three, which is only half of standard biometrics like finger or iris recognition (Gaspar, Rousselet, & Pernet, 2011). Having subjects' identity at hand, whole scalp ERP classification showed a 100% accuracy in discriminating between participants (Ruiz‐Blondet, Jin, & Laszlo, 2017). More recently, spectral power derived from MEG was shown to have participant specific embeddings dependent on sidekick cell adhesion molecule 1 encoded by the SDK1 gene, allowing discrimination and identification even between twins (Leppäaho et al., 2019). As technology on linking information and singling out individuals from large data sets is evolving, we recommend following the precautionary principle, considering any brain‐related data as personal data and consequently following the appropriate regulations. Future work will also consider linking consent to resources such as the Open Humans Project https://www.openhumans.org, which enables personal data stores. Individuals are in control of sharing their data, with whom, and for what reason. By aggregating individual data from different sources, such resources increase the richness of any data for scientific analyses while preserving privacy and allowing for consented access. The Open Brain Consent project provides a comprehensive starting point for resources that account for legal sharing of data by providing consent template forms compliant with different regulations.

## CONFLICT OF INTERESTS

We declare no conflict of interest related to this work.

## Data Availability

All material used here is distributed freely under CC‐BY license.
